# The efficacy of systemic antibiotics as an adjunct to surgical treatment of peri-implantitis: a systematic review

**DOI:** 10.1186/s12903-021-02020-1

**Published:** 2021-12-27

**Authors:** Malene Øen, Knut N. Leknes, Bodil Lund, Dagmar F. Bunæs

**Affiliations:** 1 Department of Clinical Dentistry – Periodontics, Faculty of Medicine, University of Bergen, Aarstadveien 19, 5009 Bergen, Norway; 2 Division of Oral Diagnostics and Oral Rehabilitation, Department of Dental Medicine, Karolinska Institutet and Medical Unit for Reconstructive Plastic- and Craniofacial Surgery, Karolinska University Hospital, Stockholm, Sweden

**Keywords:** Humans, Peri-implantitis, Dental implant surgery, Systemic antibiotics, Systematic review, Therapy

## Abstract

**Background:**

Microbial biofilm accumulation is the main cause of peri-implantitis. The majority of surgical peri-implantitis treatment protocols suggests adjunctive use of systemic antibiotics to target specific putative bacteria. The aim of this systematic review was to critically evaluate the adjunctive use of systemically administered antibiotics in surgical treatment of peri-implantitis by reviewing previously published systematic reviews and primary studies.

**Methods:**

A systematic literature search was conducted in four electronic databases (MEDLINE, The Cochrane Library, EMBASE, and Web of Science) for randomised controlled trials, cohort studies, case–control studies, and systematic reviews reporting surgical treatment of peri-implantitis with and without adjunctive systemically administered antibiotic therapy. The included systematic reviews and primary studies were qualitatively assessed using AMSTAR and GRADE, respectively. No restrictions were set for date of publication, journal, or language.

**Results:**

The literature search identified 681 papers. Only seven systematic reviews and two primary studies met the inclusion criteria. Four out of seven included systematic reviews concluded that no evidence exists for use of systemic antibiotics to improve the clinical outcomes in surgical treatment of peri-implantitis. One review did not estimate the level of evidence, one did not clearly state any beneficial effect, whereas one reported a limited adjunctive effect. Further, the two included primary studies did not show a long-term significant benefit of adjunctive use of systemically administrated antibiotics. However, one study reported a short-term adjunctive effect in patients with modified surface implants. Due to heterogeneity in study design, low number of included primary studies, and grade of bias, no meta-analysis was performed.

**Conclusion:**

The use of systemically administered antibiotics as an adjunct to surgical interventions of peri-implantitis cannot be justified as a part of a standard treatment protocol. A pervasive problem is the lack of uniform diagnosis criteria for peri-implantitis, deficient information about patient characteristics, absence of high quality long-term randomised controlled trials, and authors’ declaration on conflict of interest.

**Supplementary Information:**

The online version contains supplementary material available at 10.1186/s12903-021-02020-1.

## Background

The prescription of systemically administered antibiotics for the prevention of postsurgical complications and/or beneficial surgical outcome effects, has remained a controversial subject for decades [1–4]. Original protocols for implant placement advocated dogmatic or consensus-based antibiotics prophylaxis and prolongation of antibiotic treatment during the postoperative period [5]. A recently published systematic review concluded that there is no benefit of antibiotic prophylaxis on implant survival in uncomplicated implant surgery in healthy patients [6].

Peri-implantitis is a pathological condition occurring in tissues surrounding dental implants, characterized by inflammation of the peri-implant mucosa and subsequent progressive loss of alveolar bone [7]. This infectious condition affects about two out of 10 patients [7–9]. With an estimation of 12 million implants placed annually worldwide by an increasing number of clinicians with varying expertise, there are concerns that peri-implantitis is a growing complication within dentistry [10]. Depending on the case definition applied [11], prevalence of peri-implantitis ranges from 10–22% at implant level [12] and 22–45% at patient level [13, 14].Typically, the development of peri-implantitis appears within the first few years after the implant has been functionally loaded [9].

Numerous peri-implantitis treatment protocols have been advocated, including non-surgical, surgical and combined approaches. Non-surgical treatment alone has shown unpredictable treatment outcomes [15, 16], while long-term data on outcomes following surgical treatment show only minor bone level gain [16, 17]. Nevertheless, access surgery is considered an essential part of the therapy with an impeding effect on the progression of peri-implantitis [16–18]. A “gold standard” protocol across the general population or in specific patient groups, has not yet been identified [18, 19]. Surveys of registered specialists in periodontology in Australia, the United Kingdom, and the United States confirm an absence of consensus and standardized therapeutic protocols [20, 21]. One explanation might be lack of high-quality studies limiting the opportunities to perform a meta-analysis on the effects of peri-implantitis treatment [18].

Over decades, it has been assumed that management of peri-implantitis could be adopted from treatment protocols and guidelines for periodontitis. In systemically healthy adult periodontitis cases, adjunctive use of systemic antibiotics is not justified due to minor infection risk following periodontal surgery [22–24]. Systemic antibiotic as part of systematic periodontal therapy is recommended only in immunocompromised cases or in cases with aggressive or non-responding periodontitis [22, 23]. Surprisingly, the majority of surgical peri-implantitis treatment protocols suggests adjunctive use of systemic antibiotics to target specific putative bacteria [16, 18, 25]. One argument for adjunctive usage, is that peri-implantitis infections are not confined to the connective tissue compartment and potentially could spread to bone marrow area [26]. Because of diverse microbiomes in peri-implantitis lesions, the use of broad-spectrum antibiotics might be necessary [25]. The drawbacks are not only increased risk of altering normal protective microflora, superinfections, and allergic reactions, but also to potentiate antibiotic resistance [27–29].

As there seems to be a lack of controlled clinical trials evaluating the efficacy of systemic antimicrobial therapy as an adjunct to surgical peri-implantitis therapy [30], additional outcome effect still remains questionable [16, 17, 29, 31, 32]. The scarce scientific documentation together with a shortage of treatment guidelines, may lead to excessive usage during surgical treatment of peri-implantitis [28]. Thus, the aim of the present systematic review was to provide knowledge by revisiting the available scientific literature through a two-stage approach consisting of quality appraisal of relevant systematic reviews and assessment of primary studies to critically evaluate the efficacy of systemically administered antibiotics as an adjunct to surgical treatment of peri-implantitis.

## Methods

The study protocol was registered in the PROSPERO database (International Prospective Register of Systematic Reviews hosted by the National Institute of Health Research, University of York, Centre for Reviews and Dissemination) with the identification number CRD42020134989 [33]. The manuscript is prepared according to preferred Reporting Items for Systematic Reviews and Meta-Analysis (PRISMA) guidelines [34].

### Focused question

For the identification of relevant studies, the present review aims to address the following focused question: “Does systemically administered antibiotics improve treatment outcomes following surgical intervention of peri-implantitis?”

### Criteria for considering studies (PICOT) [35]

The following Participants, Intervention, Comparison, Outcomes, and Time (PICOT) framework was employed to guide the inclusion and exclusion of studies for the focused question:

- Participants (P): Patients with peri-implantitis who underwent surgical treatment
- Intervention (I): (A) Surgical treatment of peri-implantitis. (B) Head-to-head comparison of different therapeutic antibiotic compounds or regiments
- Comparison (C): Surgical treatment of peri-implantitis with and without systemically administered antibiotics, placebo, or other non-antibiotic treatment such as antiseptics rinsing
- Outcome (0):
  *Primary *outcome: Radiographic marginal bone level.
  *Secondary outcomes*: Bleeding on probing (BoP)/suppuration on probing (SoP), probing depth (PD), pain, implant loss, microbial composition, and quality of life (QoL)
- Time (T): Minimum follow-up of 3 months after surgical intervention

### Eligibility criteria

To be eligible for inclusion, studies had to fulfil the following inclusion criteria:

- Reporting randomized controlled trial (RCT), cohort study, case–control study, or systematic review
- Include patients being surgically treated for peri-implantitis with and without adjunctive systemically administered antibiotic therapy
- Abstract available in English language

The following exclusion criteria were applied:

- In vitro or animal studies
- Case reports
- A follow-up less than 3 months
- Non-surgical peri-implantitis treatment
- Use of prophylactic antibiotics
- No clear definition of peri-implantitis
- Systematic reviews that were followed by more recent systematic reviews by the same author, non-systematic reviews, treatment guidelines, letters, position papers, and consensus statements

### Search strategy

A detailed systematic literature search was undertaken by one author (MØ) and one expert reviewer at Medical Library at University of Bergen using the following four electronic databases: The National Library of Medicine (MEDLINE via PubMed), the Cochrane Central Register of Controlled Trials (The Cochrane Library, Wiley), EMBASE via OVID, and Web of Science. Hand-searching included reviewing citation lists of the retrieved full-text articles, and index search in three different journals (Journal of Periodontology, Journal of Clinical Periodontology, and Clinical Oral Implants Research). To detect more recent publications a complementary search was performed September 2021 in the four databases using the same search strategy as in the main search. No restrictions were set for date of publication, journal, or language.

The protocol for the bibliographic research was made on MeSH terms and free text words combined through Boolean Operators (AND, OR). Selection criteria were broad during identification and screening to decrease search specificity (anticipating low agreement between investigators, thus decreasing the risk of omitting relevant articles) and specific during inclusion to increase search precision. The primary search strategy was constructed based on two domains (“peri-implantitis”/“periimplantitis”) AND (“anti-bacterial agents”/ “anti-infective agents”). The search strategy for the various database is summarized in Additional file 1: Table S1.

### Study selection

The retrieved list of publications was reviewed by MØ for a crude exclusion of irrelevant publications based on title. In case of uncertainty, a study was retained until next selection step. The remaining titles were screened by all four authors as a group. The abstracts were distributed among the four authors (MØ, KNL, BL, and DFB) who independently screened included abstracts and selected articles that met the inclusion/exclusion criteria. In case of uncertainty, the abstract was read by all four authors and a decision about study eligibility was reached through discussion and consensus among the reviewers. Eligible studies were included in the second round and allocated into primary studies and systematic reviews, which were read in full text independently in duplicates by two teams, respectively MØ/DFB and BL/KNL. Only studies fulfilling the inclusion criteria were reviewed and considered for data extraction. At each stage, disagreement between reviewers were solved through discussion and consensus; if a disagreement persisted, a third reviewer settled the discussion.

### Data extraction and method of analysis

The goal of the quantitative assessment was to evaluate and compare the changes in QoL, pain, implant loss, microbial composition, patient characteristics, treatment approaches, and different outcome variables such as radiographic marginal bone level, BoP/SoP, and PD. A predetermined data extraction form based on the aforementioned criteria was used to record data from each included study. However, because of a lack of sufficient data on QoL and pain from the included RCTs, only quantitative analyses on the other variables were conducted. A meta-analysis was not performed because only two primary studies were included, both with moderate risk of bias, displaying heterogeneity in study designs and outcome variables [36].

### Quality assessment

The risk of bias of the systematic reviews was assessed using AMSTAR [37]. The reviews were classified according to the criteria showed in Additional file 2: Table S2 as low, moderate, or high risk of bias. The level of bias in the primary studies was assessed using GRADE [38], listed in Table 1.

**Table 1:**
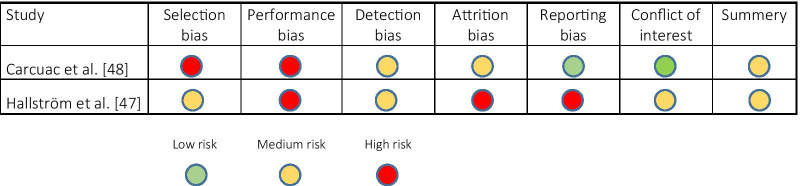
Outcome of quality assessment of the primary studies using GRADE

Two teams of reviewers independently evaluated the overall study quality and risk of bias of included reports, using all the checklist items of the respective scales. The articles were graded by the teams. In case of disagreement, consensus was achieved after discussion with the other team.

## Results

### Literature search

**Fig. 1:**
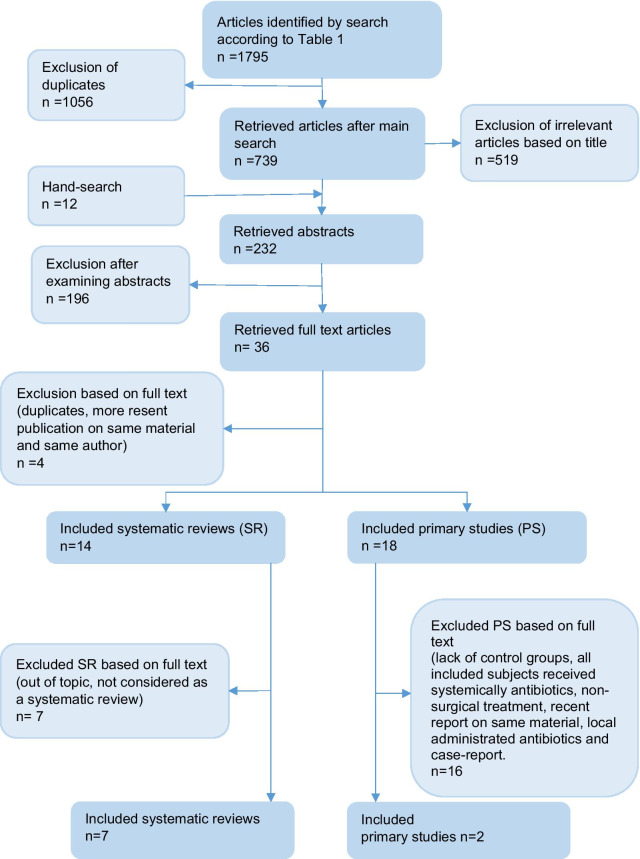
Flow chart representing study selection and inclusion

The initial literature search identified a total of 681 articles after de-duplication. The systematic search flow is outlined in Fig. 1. Twelve articles were retrieved through hand-search, whereas no additional articles were found in the grey literature search. Following first-stage screening of titles and abstracts and removal of additional duplicates, 36 articles qualified for full-text screening. Four articles were excluded due to a more recent publication based on the same patient material. Most of the studies included in the primary search was excluded due to lack of control group, being animal studies or testing other forms of peri-implantitis treatment strategies. Altogether 32 studies were included, of which 18 were primary studies and 14 systematic reviews.

### Study selection and qualitative assessment

#### Systematic reviews

##### Study selection

Following full text reading of the 14 systematic reviews, seven were included (Table 2). The main reasons for exclusion were “out of topic” [39–43], or “not considered as a systematic review” [28, 44]. Table 2 summarizes included and excluded systematic reviews.

**Table 2:** Included and excluded systematic reviews

References	I	E	Reason for exclusion
Chan et al. [39]		X	Out of topic
Esposito et al. [80]		X	Out of topic
Heitz-Mayfield and Lang [31]	X		
Heitz-Mayfield and Mombelli [18]	X		
Klinge et al. [32]	X		
Keeve et al. [17]	X		
Kotsovilis et al. [41]		X	Out of topic
Mombelli et al. [46]	X		
Naujokat et al. [42]		X	Out of topic
Renvert et al. [16]	X		
Roccuzzo et al. [45]	X		
Schou et al. [44]		X	Not a systematic review
Schwarz et al. [43]		X	Out of topic
Verdugo et al. [28]		X	Not a systematic review

Year, year of publication; I, Inclusion; E, exclusion

##### Quality assessment and data extraction

The quality assessment of the included systematic reviews identified two reports with moderate risk of bias due to lack of information about patient characteristics [18] and conflict of interest [45]. Five were considered as high risk of bias [16, 17, 31, 32, 46] because of shortcomings in study selection (missing flow chart or steps in recommended search strategy for systematic reviews and reporting that the quality assessment was made by only one researcher). No studies were deemed at low risk of bias. Table 3 summarizes the risk of bias in the included systematic reviews.

**Table 3:** Data extraction and risk of bias in the systematic reviews

References	Objective	Main results^a^	Estimated level of evidence	Knowledge/knowledge gaps^a^	Level of risk of bias comments	Comments
Heitz-Mayfield and Lang [31]	To review antimicrobial therapy, including the use of antiseptic and/or antibiotic agents, administered locally or systemically for the treatment of peri-implant diseases	The antibiotic regiments used varied between studies with respect to type of antibiotic, dosage, delivery system, duration, and commencement of antibiotic therapy. Adverse effects related to the antimicrobial agents and patient compliance were not considered. There is insufficient evidence to recommend a particular anti-infective protocol for the treatment of peri-implantitis. n = 5	Evidence is lacking	Treatment of peri-implantitis with regenerative surgery and systemic antibiotics improved the clinical situation. No controls were included. The relative importance of included treatment measures cannot be determined	High risk of bias. No meta-analysis was performed	
Heitz-Mayfield and Mombelli [18]	To evaluate the success of treatments aimed at the resolution of peri-implantitis in patients with osseointegrated implants	The length of follow-up varied from 3 months to 7.5 years. Due to the heterogeneity of study designs, peri-implantitis case definitions, outcome variables, and reporting, no meta-analysis was performed. Successful treatment outcomes at 12 months were in 0% to 100% of patients treated in 9 studies and 75% to 93% of implants treated in 2 studies	The available evidence does not allow any specific recommendations for the therapy of peri-implantitis	All studies included had either an unclear or high risk of bias. Peri- or postoperative systematic antibiotics seem to be beneficial	Moderate risk of bias. No meta-analysis was performed	
Klinge et al. [32]	To systematically assess the efficacy of anti-infective therapy as a component of the treatment of peri-implantitis	The outcomes following anti-infective treatment of peri-implantitis are highly variable. No data available to support specific treatment protocols	No evidence exists on the significance of anti-infective treatment for the longevity of the implant	Improved clinical parameters were reported. However, a non-medicated control group was not included	High risk of bias. No meta-analysis was performed	
Mombelli et al. [46]	To review the literature investigating the results of surgical treatment of peri-implantitis in man	All current approaches include the elevation of a mucoperiosteal flap and the removal of the peri-implant inflammatory granulation tissue. The majority of protocols include the systemic administration of antibiotics and chlorhexidine rinses. n = 33	The available evidence does not allow any firm recommendations for the surgical therapy of peri-implantitis	Evidence for true osseo-reintegration onto previously contaminated implant surfaces is non-existent for naturally occurring human peri-implantitis	High risk of bias. No meta-analysis was performed	
Renvert et al. [16]	To review surgical therapy for the control of peri-implantitis	Decontaminating flap surgery decreases peri-implantitis. Laser treatment has no beneficial effect. Placement of bone or bone substitutes may fill peri-implantitis defects	Level of evidence not estimated	Effect of adjunctive antibiotics. Additional effect of membrane placement	High risk of bias	Study selection and data extraction not performed by two independent persons. Included and excluded studies not accounted for. Characteristics and quality assessment of included studies lacking. Conflict of interest not stated
Roccuzzo et al. [45]	To review clinical outcome of peri-implantitis and supportive treatment	Peri-implantitis treatment followed by supportive care resulted in high success rates in the medium to long term	Not clearly stated	Not defined	Moderate risk of bias	Conflict of interest not stated
Keeve et al. [17]	To review surgical treatment of peri-implantitis with non-augmentative techniques	Clinical parameters can be reduced by surgical non-regenerative treatments. Implantoplasty is more efficient than systemic antibiotics. No long-term effect of local chemical irrigation	Limited	Not clearly stated	High risk of bias	Hand search of literature not performed. Excluded studies not accounted for. Patient characteristics of included studies not shown

^a^According to authors

##### Estimated level of evidence

Four out of seven included systematic reviews concluded that no evidence exists indicating that systemic antibiotics improved the clinical outcomes of surgical treatment of peri-implantitis [18, 31, 32, 46]. One review did not estimate the level of evidence [16], one did not clearly state any beneficial effect [45], whereas one reported a limited adjunctive effect [17].

#### Primary studies

##### Study selection

A total of 18 primary studies were read in full text. Only two studies met the inclusion criteria [47, 48]. The main reasons for exclusion were lack of control groups [49–54], and/or that all included subjects received systemic antibiotics [55–60]. One study was excluded as a more recent report was published on the same material [61], one reported outcomes of non-surgical treatment [62], one adjunctive use of locally administered antibiotics [52], whereas one was a case report [63]. Table 4 summarizes included and excluded primary studies.

**Table 4:** Included and excluded primary studies

References	I	E	Reason for exclusion
Bianchini et al. [49]		x	No control group
Buchter et al. [62]		x	Non-surgical and local antibiotic treatment
Carcuac et al. [48]	x		
Carcuac et al. [61]		x	Newer publication on same material
Cha et al. [83]		x	Local antibiotic treatment, all subjects got postsurgical. antibiotics
Charalampakis et al. [50]		x	No control group
Hallström et al. [47]	x		
Heitz-Mayfield et al. [25]		x	All subjects got antibiotics
Heitz-Mayfield et al. [56]		x	All subjects got antibiotics
Jepsen et al. [23]		x	All subjects got antibiotics
Khoury and Buchmann [51]		x	No control group
Maximo et al. [52]		x	No control group
Mercado et al. [53]		x	No control group
Mombelli and Lang [58]		x	All subjects got antibiotics
Roos-Jansaker et al. [59]		x	All subjects got antibiotics
Serino and Turri [54]		x	No control group
Verdugo [63]		x	Case-report
Roccuzzo et al. [60]		x	All subjects got antibiotics

Year, year of publication; I, Inclusion; E, exclusion

In the case of two publications reporting on the same cohort at different follow-up intervals, it was decided to pool all relevant details as a single report providing more comprehensive data for inclusion in the qualitative and quantitative analyses. Insufficient volume of quantitative outcome data from only two included RCTs, precluded the use of meta-analysis [64].

##### Quality assessment and data extraction

Quality assessment of the two included studies rendered an estimated medium risk of bias for both [47, 48]. The main features of the two studies are shown in Table 5; population characteristics; peri-implantitis case definition; surgical protocol; and supportive care during follow-ups.

**Table 5:** Data characteristics in the primary studies

References	Study	Method (Study design)	Follow-up time	Disease definition	Participants, implant at baseline	Measurements	Pre-treatment	Treatment
Carcuac et al. [48]	Surgical treatment of peri-implantitis: 3-year results from a randomized controlled clinical trial	RCT	36 months	PD > = 6 mm, BoP/SoP and marginal bone loss > 3 mm	Patients n: 100, Implants n: 179	PD reduction, Radiographic bone level, BoP, Implant loss	Not mentioned	Pocket elimination using resective techniques
Hallström et al. [47]	Open flap debridement of peri-implantitis with or without adjunctive systematic antibiotics: A randomized clinical trial	RCT	12 months	Peri-implant marginal bone loss ≥ 2 mm as determined from a comparison of the bone level 1 year following implant reconstruction with the bone level at screening, or ≥ 3 mm in depth as determined from peri-apical radiograph, in combination with probing depth ≥ 5 mm and with bleeding or suppuration on probing	Patients: n = 39, Implants: n = 39	PD reduction, Radiographic bone level, microbial composition and load	Not mentioned	Removal of inflamed tissue and mechanical cleaning of the implant without any additional therapy

References	Procedures in experimental group/sites	Procedures in control group/sites	Additional surface treatment	Antimicrobial treatment	Maintenance	Implant type	Patient characteristics (smokers, systemic diseases, other modification factors)	
Carcuac et al. [48]	Group 1 and 2: systemic antibiotics (amoxicillin 2 × 750 mg daily)	Group 3 and 4: No other treatment	Group 1 and 3: mechanical implant surface decontamination supplemented by an antiseptic agent (0.2% chlorhexidine gluconate) Group 2 and 4: mechanical implant surface decontamination with saline	Not mentioned	First year every 3 months, after that by referring dentist. Control at 12 and 36 months	Nobel Biocare (turned): 43 Nobel Biocare (TiUn): 87 Astra Tech (TiO): 9, Astra Tech (OsseoSpeed): 24, Straumann (SLA): 13, Neoss(ProAct): 3	Current smokers: 33% (33) History of periodontitis 84% (84), Diabetes 5% (5)	
Hallström et al. [47]	Peri- treatment Zithromax 250 mg × 2 at the day of surgery, 250 mg × 1 per day during four additional days	No other treatment	Cotton gauze in saline and cleaning with sterile curettes	Chlorhexidine 0.2% × 2 daily both groups	Every 3 months	Brånemark (t:5 c:11), Astra Tech (t:7 c:5), Straumann (t:7 c:3, Cresco (t:1)	Smoking history: 36.7% (14) of the participants, current: 30.7% (12) (t 40% (8) c 21% (4))	

BL, baseline; t, test; c, control; PD, probing depth; SoP, suppuration on probing; BoP, bleeding on probing

Carcuac et al. compared surgical treatment of peri-implantitis with and without adjunctive use of amoxicillin in four groups of patients [48]. They followed 100 subjects with 179 implants over 3 years, with a participant mean age of 66.3 (21–90 years). In patient group 1 and 2, a 10-d systemic antibiotic regimen (amoxicillin 2 × 750 mg daily) started 3 days prior to surgery. Patients in group 3 and 4 did not receive systemic antibiotics. Hallström et al. compared treatment outcomes following surgical treatment with and without adjunctive use of Zithromax® (Sandoz AS, Copenhagen, Denmark) [47]. They followed 39 subjects with 39 implants for 1 year, with a participant mean age of 70.5 (26–86 years). The test group received Zithromax® 250 mg × 2 at the day of surgery, and 250 mg × 1 per day during 4 additional days. The 19 patients in the control group were treated with open flap debridement, whereas the 20 test individuals received open flap debridement and systemic antibiotics.

For both studies [47, 48], the treatment was performed by trained periodontists in a university stetting or at a hospital, but none of the studies provided detailed information about a supportive care protocol. The evaluations of each bias item for the included studies are summarized in Table 1.

##### Outcome

Additional file 3: Table S8 summarizes the effects of adjunctive use of systemic antibiotic following surgical treatment of peri-implantitis. From baseline to 3 years, Carcuac et al. reported a radiographic bone level gain of 0.32 ± 1.35 mm in the groups receiving systemic antibiotics (AB+), whereas a radiographic bone level loss of 0.51 ± 1.87 was observed in the non-antibiotic groups (AB−) [47]. However, the positive effect of systemic antibiotics faded after 1 year. From year 1 to year 3, a minor mean bone loss was detected in all four groups: Group 1 and 2 (AB+) and Group 3 and 4 (AB−) 0.21 ± 0.94 and 0.06 ± 1.37, respectively. Compared with baseline, increased PD (> 5 mm) was noted at 35% of the implants at 3 years. Systemic antibiotics decreased the probability for PD > 5 mm at implants with modified surfaces from 58 to 34%. An opposite effect was observed at non-modified implants (AB−: 9%; AB+: 22%). At 3 years, systemic antibiotics had no effect in terms of reduced BoP/SoP.

Of 121 implants included, 69% were successfully treated according to the authors (no bone loss > 0.5 mm). Totally, 20 implants were explanted (six at year 1) during the observation period. Predicted probability of a successful outcome varied between 91% (AB−) and 89% (AB+) for non-modified surface implants and between 32% (AB−) and 78% (AB+) for modified surface implants. Benefits of systemic antibiotics were limited to implants with modified surfaces (0.3 mm (AB+) and − 1.3 mm (AB−) and to the first year of follow-up [48].

At 1 year, Hallström et al. reported a non-significant bone level gain of 0.6 mm in patients receiving systemic antibiotics (+ 0.6 mm) and 0.4 mm in the control group without systemic antibiotics (+ 0.4 mm) [47]. In this study, successful clinical outcome was defined as PD ≤ 5 mm, no suppuration, no BoP at the implant site, and bone loss ≤ 0.5 mm at 1 year. A successful study outcome was identified in seven individuals (46.7%) in the test and four individuals (25%) in the control group. Carcuac et al. did not define successful clinical outcome, but analysed clinical and radiographic changes at baseline, at 1 and 3 years [47]. None of the studies evaluated patient-related outcomes. Sample size calculation to estimate the minimal number of individuals needed to detect a significant positive treatment outcome between groups, was only reported in the Hallström’s study.

## Discussion

### Summary of main results and clinical relevance

By reviewing original studies and systematic reviews, the present review shows that there is at best, limited evidence for a sustained adjunctive effect of systemically administered antibiotics in surgical treatment of peri-implantitis. Systemic antibiotics might be beneficial as adjunct to surgical treatment in specific group of patients and implants with specific surface modifications. Previous clinical studies have major shortcomings as lack of information about patient characteristics, absence of high-quality long-term (> 3 years of follow-up) RCTs, and authors’ declaration on conflict of interest. The impact of these shortcomings is discussed in the present systematic review.

An included systematic review from 2002, concluded that the evidence for a consistent and clinically relevant advantage using systemically administered antibiotics can be questioned [32]. Special attention was drawn to the problem with no standardized protocol in treatment of peri-implantitis and the lack of RCT’s with a low grade of bias. This perception is supported by all included systematic reviews, and particularly highlighted in the latest review from 2019, stating that there is a need for well-designed RCTs with sufficient power to evaluate surgical non-regenerative treatment of peri-implantitis [11]. When pooling data from the seven included systematic reviews, 69% of the included primary studies used systemically or locally administered antimicrobial therapy as a part of the surgical treatment protocol of peri-implantitis. This documents that adjunctive use of antimicrobials is a general trend more than an exception.

In the included primary studies, there were limited information about patient characteristics. Hallström et al. registered smoking status [47], but neither Carcuac et al. nor Hallström et al. discussed the potential impact of patient characteristics on the treatment outcomes [47, 48]. On the other hand, Carcuac et al. reported that implant surface characteristics may impact peri-implantitis susceptibility and resolution of inflammation [48]. At 3 years, treatment success was more frequent observed at implants with a non-modified (“turned”) surface compared with implants with modified surface [48]. This indicates that the effect of adjunctive systemic antibiotics in the surgical treatment of peri-implantitis may be dependent on patient as well as implant surface characteristics.

In 2016 The World Health Organization (WHO) decided to prioritize the development of evidence-based recommendations for use of antibiotics in surgical therapy [65]. WHO does not recommend prolonged surgical antibiotic prophylaxis after completion of the surgery for the purpose of preventing site infections. The recommendation of these drugs in oral medicine should follow the same precepts as those of general medicine [22]. However, lack of treatment guidelines, slow adoption of guidelines, a varied skill set of the average dentist, and pressure from the patients, might contribute to unnecessary antibiotics prescriptions [66]. The awareness among dentists and administrative personnel of their responsibility for the increasing antibiotic resistance should therefore be highlighted.

Management of antimicrobial resistance requires the implementations of two processes: infection control practices to limit spread of resistant microorganisms and hospital policies of good antimicrobial use stewardship, which may include antimicrobial usage control [67]. Generally, multidrug resistant bacteria are a growing global concern most likely caused by frequent and inappropriate use of antibiotics [68–71]. Data from the United States show that up to 60% of the microorganisms isolated from infected surgical wounds was antibiotic resistant [65]. Concerns have also been raised that an extensive use of systemic antibiotics in periodontal therapy, particularly when administered to counterbalance incomplete mechanical instrumentation or poor oral hygiene, could contribute substantially to the development of bacterial antimicrobial resistance [72–74]. Similar concerns should be expressed to peri-implantitis treatment protocol comprising systemically administered antibiotics. A recently published systematic review found that most of the 18 included primary studies did not report full-mouth plaque scores [45]. Nevertheless, 13 of the 18 studies used systemically administered antibiotics as part of the surgical treatment protocol.

### Comparison with other studies

Treatment of peri-implantitis aims to re-establish and maintain peri-implant health around implants with reduced bone support [75]. The main goal is to mechanically remove or disturb the bacterial biofilm contaminating the exposed implant surface. Long-term data attained from the included systematic reviews, reveal only minor changes in quantitative parameters such as BoP, SoP, radiographic bone level gain, and reduction in PD following systemically administered antibiotics as an adjunct to surgical treatment of peri-implantitis. Studies reporting superior treatment outcomes are exceptions, and their quality assessments have categorized the risk of bias as high [18, 31].

None of the included systematic reviews discussed microbiological changes before and after surgical treatment and only one of the primary studies investigated bacterial composition. Hallström et al. failed to find any statistical differences in bacterial load between the two treatment groups in their RCT [47]. Trends of decreasing bacterial loads were found between baseline, 2 and 4 weeks in both the experimental and the control group. Regarding periodontitis, Haffajee found that the short-term benefit of adjunctive systemic antibiotic treatment was not critical for the long term survival of teeth [72]. A bone gain of 0.3 mm would be equivalent to reversing 4–7 years of disease progression in a well-maintained population. Similarly, the included RCTs and systematic reviews indicate that on average, systemically administered antibiotics contribute to therapeutic short time success in the treatment of peri-implantitis but fails to generate the long-term benefit.

The wide heterogeneity of peri-implant disease definition has also been a problem in the field of peri-implantitis research. All included systematic reviews reported wide heterogeneity of peri-implant disease definition. Until World Workshop of Periodontitis 2017 [9], no uniform clinical definition of peri-implantitis existed. Although bone loss and clinical manifestation of inflammation are the most important diagnostic criteria of peri-implantitis, a significant crestal bone loss over time must be verified to distinguish between peri-implant mucositis and peri-implantitis. The large variations in case-definitions make it inapplicable to compare different study outcomes following treatment of peri-implantitis. This partly reflects the wide heterogeneity of conclusions in former systematic reviews and lack of treatment standards founded in meta-analysis [76]. The majority of the included systematic reviews highlight this point in the discussion and conclusion section.

### Strength and limitation of the study

A common denominator trough all included systematic reviews was that no conclusion could be drawn due to lack of high-quality studies. The present review specified inclusion criteria as minimum follow-up time of 3 months, presence of control group, and low or moderate level of bias. The primary search included 681 studies on surgical treatment of peri-implantitis. Of these, only two RCTs fulfilled the criteria [47, 48]. The lack of high-quality studies might be related to high research costs and ethical issues when designing and testing pharmacological drugs in RCT. Research bias towards publishing positive and encouraging results is another dilemma [77].

The market for dental implants keeps growing, and the size of the global market for dental implants had a value of USD 3.6 billion in 2020 [78]. Interestingly, the main sponsorship of major national and international conferences has been carried out by implant and biomaterial industries [79]. The leading implant companies may focus on functionality rather than biological complications and environmental dilemmas with overuse of antibiotics. Detailed information on potential conflict of interest and sponsorship are pivotal for an adequate understanding and appropriate interpretation of the reported study outcomes. Not only financial conflict of interest (COI), but also academical. In five of the included systematic reviews a COI statement was not included. One systematic review found that only four of 18 included studies reported on funding [45]. Another review of RCT`s, showed that seven out of nine included studies were funded by industry directly involved in the product being tested; one study did not receive funding, and one reported unclear funding [80]. The prevalence of COI statements/sections seems to be more underreported (32.1%) in dental journals compared with other medical journals (13.6%) [81]. However, a systematic review that compared sponsored and non-sponsored RCT assessing different implant systems, found no significant difference in marginal bone loss related to sponsorship status [82].

Finally, some additional study limitations should be acknowledged. (1) Lack of uniform diagnostic criteria. (2) Different implant surface modifications. (3) Variation in length of follow-up and treatment modalities. (4) The included RCTs are from the same country, published the same year, and in the same journal. (5) Restricted inclusion criteria. RCTs were only included if the primary outcome was comparison of surgical therapy of peri-implantitis with and without adjunctive systemically administered antibiotics and if the bias rate was low or moderate. (6) Only including two RCTs resulting in data scarcity and heterogeneity precluding meta-analysis. (7) None of the included studies were considered at low risk of bias, indicating a strict interpretation in the use of GRADE and AMSTAR guidelines.

## Conclusions

The use of systemically administered antibiotics as an adjunct to surgical interventions of peri-implantitis cannot be justified as a part of a standard treatment protocol. Considering the pathological pattern of peri-implantitis, systemic antibiotics might be beneficial as adjunct to surgical treatment in specific patient groups and implants with specific surface characteristics. In these cases, benefit versus harm analysis including considerations on the overall use of antibiotics for the individual patient and public health must be considered. This systematic review shows that there is limited evidence for adjunctive use of systemic antibiotics due to scarcity of published high-quality clinical studies. Future studies evaluating the efficacy of systemically administered antibiotics as an adjunct to surgical treatment of peri-implantitis in high-quality long-term RCT are warranted.

## Supplementary Information

**Additional file 1: Table S1.** Strategy for the literature search.

**Additional file 2: Table S2.** Quality assessment of the systematic reviews.

**Additional file 3: Table S8.** Outcome variables in the primary studies.

## Data Availability

Not applicable.

## Abbreviations

PICOT: Participants, intervention, comparison, outcomes, and time
BoP: Bleeding on probing
SoP: Suppuration on probing
QoL: Quality of life
RCT: Randomized controlled trial
COI: Conflict of interest
