# Supplementary material for: The efficacy of systemic antibiotics as an adjunct to surgical treatment of peri-implantitis: a systematic review
Source: BMC Oral Health. 2021 Dec 27;21:666. doi: 10.1186/s12903-021-02020-1 (PMC8711198; doi:10.1186/s12903-021-02020-1)
Supplement: Supplementary file 1 — Additional file 1: Table S1. Strategy for the literature search. [file 12903_2021_2020_MOESM1_ESM.docx]

Table 1. Strategy for the literature search

| Database |  | Search strategy | No of references retrieved |
| --- | --- | --- | --- |
| **EMBASE** |  |  |  |
|  | #1 | periimplantitis/ | 1951 |
|  | #2 | (Peri-Implantitis OR Peri-Implantitides OR Periimplantitis OR Periimplantitides OR Peri implantitis OR PeriImplantitides). ti,ab,kw. | 2886 |
|  | #3 | #1 OR #2 | 3284 |
|  | #4 | antiinfective agent/ | 181029 |
|  | #5 | (Antibacterial OR anti-bacterial OR Antimycobacterial OR anti-mycobacterial OR bactericide* OR antiinfective* OR anti-infective or antibiotic* OR antimicrobial OR antiseptic*). ti,ab,kw. | 705450 |
|  | #6 | #4 OR #5 | 774665 |
|  | #7 | #3 AND #6 | 540 |
|  |  |  |  |
| **MEDLINE** |  |  |  |
|  | #1 | exp Peri-Implantitis/ | 1575 |
|  | #2 | (Peri-Implantitis OR Peri-Implantitides OR Periimplantitis OR Periimplantitides OR Peri implantitis OR PeriImplantitides). ti,ab,kw. | 2923 |
|  | #3 | #1 OR #2 | 3220 |
|  | #4 | exp anti-bacterial agents/ | 761447 |
|  | #5 | (Antibacterial OR anti-bacterial OR Antimycobacterial OR anti-mycobacterial OR bactericide* OR antiinfective* OR anti-infective or antibiotic* OR antimicrobial). ti,ab,kw. | 557676 |
|  | #6 | #4 OR #5 | 1037050 |
|  | #7 | #3 AND #6 | 505 |
|  |  |  |  |
| **The Cochrane Library** |  |  |  |
|  | #1 | MeSH descriptor; [Peri-Implantitis] explode all trees | 195 |
|  | #2 | (Peri-Implantitis OR Peri-Implantitides OR Periimplantitides OR Peri implantitis OR Peri Implantitides) | 378 |
|  | #3 | #1 OR #2 | 378 |
|  | #4 | MeSH descriptor: [Anti-Bacterial Agents] Explore all trees | 12539 |
|  | #5 | (Antibacterial OR anti-bacterial OR Antimycobacterial OR Anti-mycobacterial OR bactericide* OR antiinfectiv* or anti-infective OR antibiotic* or antimicrobial OR antiseptic*) | 51443 |
|  | #6 | #4 OR #5 | 52302 |
|  | #7 | #3 AND #6 | 115 |
|  |  |  |  |
| **Web of Science** | #1 | (Peri-Implantitis OR Peri-Implantitides OR Periimplantitides OR Peri implantitis OR Peri Implantitides) | 3141 |
|  | #2 | (Antibacterial OR anti-bacterial OR Antimycobacterial OR Anti-mycobacterial OR bactericide* OR antiinfectiv* OR anti-infective OR antibiotic* OR antimicrobial OR antiseptic*) | 735717 |
|  | #3 | #1 AND #2 | 585 |
