# Supplementary material for: The efficacy of systemic antibiotics as an adjunct to surgical treatment of peri-implantitis: a systematic review
Source: BMC Oral Health. 2021 Dec 27;21:666. doi: 10.1186/s12903-021-02020-1 (PMC8711198; doi:10.1186/s12903-021-02020-1)
Supplement: Supplementary file 2 — Additional file 2: Table S2. Quality assessment of the systematic reviews. [file 12903_2021_2020_MOESM2_ESM.docx]

Table 2. Quality assessment of the systematic reviews

| Level of bias | Criteria | Q |
| --- | --- | --- |
| Low | Predetermined research question and inclusion criteria established | 1 |
|  | A minimum of two independent data extractors and consensus procedure reported | 2 |
|  | At least the database MEDLINE/PubMed used | 3 |
|  | Reproducible search strategy reported |  |
|  | The publication type used as an inclusion/exclusion criterion | 4 |
|  | Included and excluded studies reported | 5 |
|  | Relevant characteristics of included studies stated | 6 |
|  | Scientific quality assessed and reported for each included study | 7 |
|  | Alignment between scientific quality of included studies and formulating conclusions | 8 |
|  | Rational and methods for pooling results reported | 9 |
|  | Publication bias estimated. Can be omitted if publication bias was unlikely and not reported | 10 |
|  | Conflict of interest stated. This item can be omitted if conflicts of interest were unlikely | 11 |
| Moderate | A yes-answer to question 1-3 and 5-8 required |  |
| High | A no-answer to any of the questions listed under moderate risk of bias |  |
|  |  |  |
| Q, AMSTAR question. Lacking report of excluded studies is accepted | |  |
