# Supplementary material for: The efficacy of systemic antibiotics as an adjunct to surgical treatment of peri-implantitis: a systematic review
Source: BMC Oral Health. 2021 Dec 27;21:666. doi: 10.1186/s12903-021-02020-1 (PMC8711198; doi:10.1186/s12903-021-02020-1)
Supplement: Supplementary file 3 — Additional file 3: Table S8. Outcome variables in the primary studies. [file 12903_2021_2020_MOESM3_ESM.docx]

| Radiographic bone level | PD reduction (mm) | BoP/SoP positive | QoL, pain | Radiographic or re-entry bone level/bone fill | Implant loss | Microbial composition | Successful treatment | Drop- out | Conclusions |
| --- | --- | --- | --- | --- | --- | --- | --- | --- | --- |
| Carcuac et al (2017) | PD changes t: -3.00 mm±2.24 c: -2.38±2.55 | Implants total: n=73(60.3%) t: n=45(66.2%) c: n=28(52.8 %) | Not reported | Implants total: 0.04± 1.64 t: -0.32±1.35 c: 0.51±1.87 | n:20 (35.8%) | Not reported | ****Outcome 1: Total 84/121 (69.4 %) implants. t: 54/68 (79.4 %), c: 30/53 (56.6%) Outcome 2: Total 67/121 (55.4 %) implants. t: 40/68 (58.8 %), c: 27/53 (50.9%) Outcome 3: Total 40/121 (33,1 %) implants. t: 20/68 (29.4 %), c: 20/53 (37.0%) | Patients: Total n=17 Loss of implant t (6) c (2), retreatment t (4) c (3), loss to follow up n=16. Implants***: total n=5. Loss of implants n=14, surgical retreated n=7, loss to follow-up n=30 | Surgical treatment of peri-implantitis is effective. Potential benefits of systemic antibiotics are not sustained over 3 years |
| Hallström et al (2017) | Total, PD changes, mean: n=-2.73±2.39 t: -3.00±2.24 c: -2.38±2.12 |  | Not reported | t: BL: 4.6±1.6 12 months 4.0±1,6 diff.: +0.6 mm c: BL=4.9±1.7, 12 months 4.5±1.5 diff.: +0.4 mm | n:0 | 12 months: 25.8 % of the individuals presented with no detectable level of bacteria* associated with peri-implantitis. No difference between the group was detected. But increase between baseline, week 2 and 4. | ** Total 11/31 (35.5 %) individuals, t:7(46.7%) c: 4(25) | t: lost to follow up n=3, discontinued intervention n=2, c: lost to follow up n=1, discontinued intervention n=2. Total n=8 | Surgical treatment of peri-implantitis with adjunctive treatment with azithromycin did not provide 1-year clinical benefits in PD changes, microbial changes or for the combined treatment outcome assessment in comparison with open flap debridement alone |
| BL,baseline; t,test; c,control; QoL,Quality of life; PD,pocket depth.  *Aggregatibacter actinomycetemcomitans Y4, Campylobacter gracilis, Campylobacter rectus, Campylobacter showae, Helicobacter pylori, Haemophilus influenzae, Porphyromonas gingivalis, Staphylococcus aureus, Staphylococcus anaerobius, Streptococcus intermedius, Streptococcus mitis, Tannerella forsythia, Treponema denticola and Treponema socranskii  ** PPD ≤ 5 mm, no suppuration, no BOP at implant site, bone loss ≤0.5 mm between baseline and 1 year  ***test/control not sorted  **** Outcome 1: No bone loss > 0.5 mm (from radigraphic baseline), Outcome 2: 1+PPD ≤ 5 mm, Outcome 3; 2+ no bleeding/suppuration | | | | | | | | | |

Table 8. Outcome variables in the primary studies
